# The Ethnology of the American Medical Profession

**Published:** 1910-10-15

**Authors:** 


### The Ethnology of the American Medical Profession.

There are, as everyone knows, many slight differences in custom, opinion, and methods between the medical professions in the United States and in Great Britain respectively. Considering how widely diverse the conditions of practice in the two countries are, it is perhaps astonishing that these differences are not greater than is in fact the case; and there is one factor in the situation which makes it more wonderful still—this is the extraordinary degree to which the American medical profession is composed of non-British nationalities. Take up any American journal and study the names of the contributors; not a half, indeed often not a quarter, of them are of Anglo-Saxon stock. German names, Spanish, Italian, Hebrew, all these abound, with a sprinkling of Scandinavian and other European nationalities; but English, Scottish, or Irish names are noticeably lacking. To be quite logical, it must be admitted that this is no proof of any preponderance of non-British elements in the American profession as a body, but merely in that section of it which contributes copiously to medical literature. At the same time it is noteworthy that the same monopoly is visible not merely in the *Annals of Surgery*, the *Journal of the American Medical Association*, the *American Journal of Obstetrics and Gynæcology*, the *Medical Record*, and similar front-rank periodicals; it is to be detected just as surely in the local medical weeklies, of which many States in the Union possess one or more. The population of the United States is, doubtless, very much more heterogeneous than that of the British Islands, and the proportion of Continental European stock is quite considerable; still, it cannot be so high as to account for the very remarkable ethnological feature of the profession there to which attention has been drawn. Whether British-descended Americans are, in fact, being ousted from the career of medicine by those of Continental extraction is a question upon which some American contemporary may possibly be able to throw light. If it is not so, then all that can be said is that their contributions to literature are extremely scanty in proportion to their numbers. It does not necessarily follow that a tendency to seek the advertisement of periodical publicity connotes higher individual professional standing or ability. But in the mass it is fairly safe to assume that prolific authorship means high professional status: that the most advanced and able members of the profession are (in the main and notwithstanding numerous individual exceptions) those whose names most frequently adorn the pages of medical literature. Of the American clinical schools best known over on this side, perhaps the Johns Hopkins staff provides the most marked exception to these observations; there English, Scottish, and Irish names are foremost, though there are even here some others of much eminence.

There is yet another ethnological difference between the medical professions in the two great English-speaking Powers. Here the proportion of Jewish practitioners is very minute; it is extraordinarily rare for any of that race to rise high in the medical profession in England, simply because there are so very few of its members in the profession at all.

In the United States, on the other hand, there is a much larger proportion—if names go for anything—and this can scarcely be explained by a larger percentage of Hebrew immigrants in the population. More probably it points to the better prospect which the American practitioner has of adequate remuneration; and there are few things more eloquent of the under-payment of medical men in Great Britain than the way in which that shrewd commercial race avoids sending its sons into medicine. In commerce, in law, even in the covenanted services of the Crown, where there is such small scope for the peculiar financial abilities of the Jew, he is prominent; but in the two professions of teaching and medicine he is conspicuous by his absence, for the obvious reason that they are the hardest-worked and the worst paid.

